# Extracellular vesicles in venous thromboembolism and pulmonary hypertension

**DOI:** 10.1186/s12951-023-02216-3

**Published:** 2023-11-30

**Authors:** Jiwei Zhang, Xiaoyi Hu, Tao Wang, Rui Xiao, Liping Zhu, Matthieu Ruiz, Jocelyn Dupuis, Qinghua Hu

**Affiliations:** 1 Department of Pathophysiology, School of Basic Medicine, Tongji Medical College, Huazhong University of Science and Technology (HUST), 13 Hangkong Road, Wuhan, 430030 China; 2 Key Laboratory of Pulmonary Diseases of Ministry of Health, Tongji Medical College, HUST, Wuhan, China; 3 Department of Pathology, Union Hospital, Tongji Medical College, HUST, Wuhan, China; 4 Department of Cardiopulmonary Circulation, Shanghai Pulmonary Hospital, Tongji University School of Medicine, Shanghai, China; 5 Department of Respiratory Medicine, Tongji Hospital, Tongji Medical College, HUST, Wuhan, China; 6 Department of Nutrition, Université de Montréal, Montreal, Canada; 7 Montreal Heart Institute, Montréal, Québec Canada; 8 Department of Medicine, Université de Montréal, Montréal, Québec Canada

**Keywords:** Extracellular vesicles, Venous thromboembolism, Pulmonary hypertension, Pathogenesis, Diagnosis, Treatment

## Abstract

Venous thromboembolism (VTE) is a multifactorial disease, and pulmonary hypertension (PH) is a serious condition characterized by pulmonary vascular remodeling leading with increased pulmonary vascular resistance, ultimately leading to right heart failure and death. Although VTE and PH have distinct primary etiologies, they share some pathophysiologic similarities such as dysfunctional vasculature and thrombosis. In both conditions there is solid evidence that EVs derived from a variety of cell types including platelets, monocytes, endothelial cells and smooth muscle cells contribute to vascular endothelial dysfunction, inflammation, thrombosis, cellular activation and communications. However, the roles and importance of EVs substantially differ between studies depending on experimental conditions and parent cell origins of EVs that modify the nature of their cargo. Numerous studies have confirmed that EVs contribute to the pathophysiology of VTE and PH and increased levels of various EVs in relation with the severity of VTE and PH, confirming its potential pathophysiological role and its utility as a biomarker of disease severity and as potential therapeutic targets.

**Graphical Abstract:**

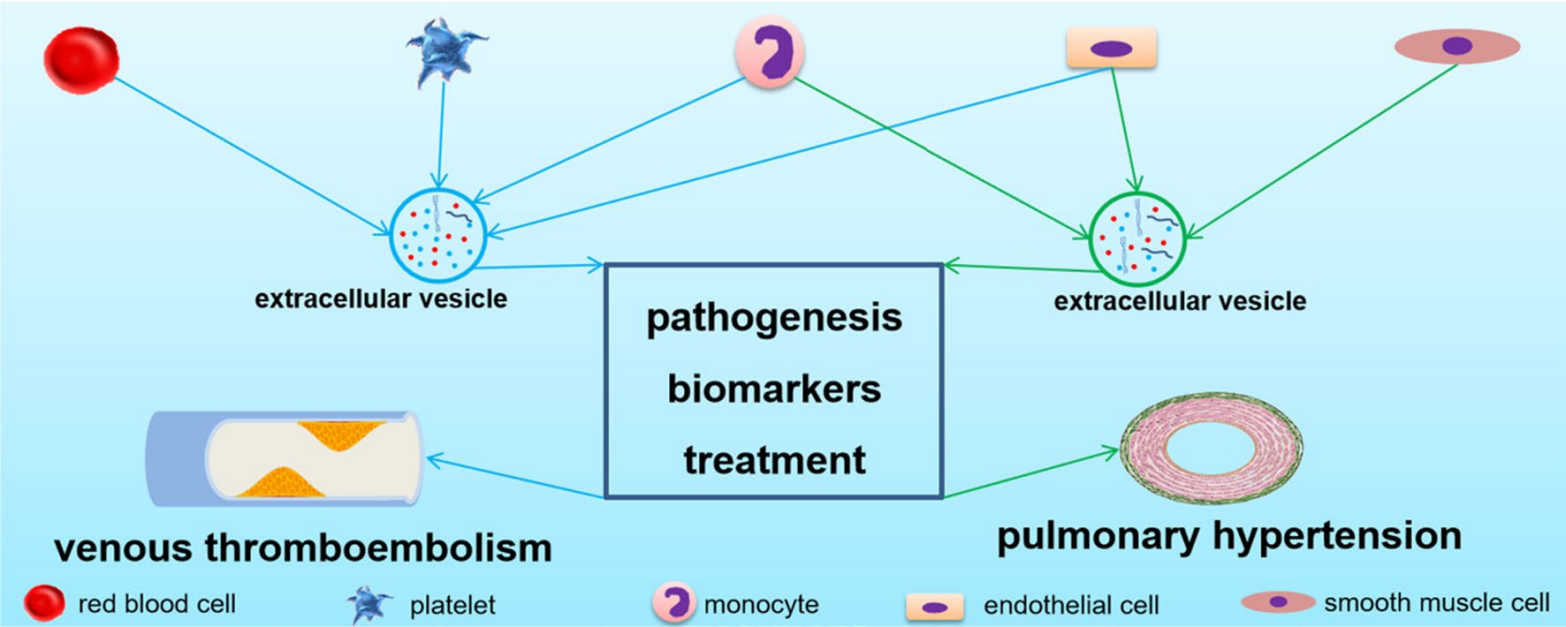

## Introduction

Extracellular vesicles (EVs) are membrane-bound structures originating from various cell types. They stem from cell membranes and cytoplasmic material and are released into the extracellular space [1]. EVs are classified into different subgroups according to their size and biogenesis [2–4]. Microvesicles (MVs) or microparticles (MPs), 0.1–1 μm in diameter, are thought to bud off directly from the plasma membrane (PM) [4]. Exosomes, less than 150 nm in diameter, are produced in multivesicular endosomes or bodies (MVBs), which can then fuse with the PM and be secreted into extracellular surroundings [2]. EVs are composed of the biological contents and surface molecules from their parent cells including nucleic acids, proteins and lipids, thereby affecting a variety of intercellular communication pathways [5, 6].

Deep vein thrombosis (DVT) and pulmonary embolism (PE), collectively referred to as VTE is a multifactorial disease [7, 8], affecting 10 million individuals each year and representing the third leading vascular disease after acute myocardial infarction and stroke [9, 10]. Virchow’s triad proposed that VTE is caused by blood hypercoagulability, vascular wall damage and impaired blood flow [11].

Pulmonary hypertension (PH) is a serious condition characterized by pulmonary vascular remodeling leading to increased pulmonary vascular resistance, ultimately resulting in right ventricle pressure overload and failure substantially reducing the duration and the quality of life [12]. Although there are many sub-types of pulmonary hypertension beyond the scope of this review, the pathophysiology is recognized as complex, involving multiple pathways and triggers with sometimes genetic predisposition as well as with a central role of endothelial dysfunction (ED) as a contributing factor [13].

VTE and PH are two distinct diseases, but they are both associated with an abnormal function of the vasculature, and chronic thromboembolic pulmonary hypertension (CTEPH) as group 4 PH is a long-term complication of unresolved PE[14]. A similarity between VTE and PH is that both conditions are associated with thrombosis in the blood circulatory system. VTE refers to primary thrombus formation in deep veins [7], while PH is caused by abnormally elevated pulmonary artery pressure due to vascular constriction and remodeling causing obliteration of the distal pulmonary vasculature with secondary associated thrombi formation [15]. In both VTE and PH, the formation of thrombosis can lead to disorders of the blood circulation system, which causes a series of severe symptoms and complications.

The distinction between the two diseases mainly includes the following aspects. First, they occur at different sites. VTE occurs mainly in the deep veins of the lower limbs and usually causes symptoms like lower limb swelling, pain and varicose veins [7]. PH, which occurs in the pulmonary blood vessels, causes symptoms such as dyspnea, chest pain and syncope [13]. Secondly, the etiology of VTE and PH also vary from each other. The main causes of VTE include prolonged immobilization, surgery, tumor and genetic factors [11]. However, the etiology of PH has greater complexity and may be related to various factors such as genetics, thromboembolism and the interactions between environmental factor(s) and human body, [13]. While the treatment of VTE consists essentially of anticoagulation, the treatment of PH requires an individualized approach including drugs that interfere with the lung vascular remodeling process. In addition, there are also differences in the prognosis of VTE and PH. Although both diseases can cause severe complications, the prognosis of PH is generally worse than VTE. PH is a chronic disease with a relatively slow progression and is more difficult to treat [15]. In contrast, VTE can often be controlled by timely anticoagulation therapy and active rehabilitation measures [7].

EVs play a key regulatory role in the occurrence and progression of VTE and PH. Thrombosis is one of the main characteristics of VTE, and EVs can carry coagulation factors and platelet-activating factors, which can promote the formation of thrombosis. EVs can activate endothelial cells and platelets, increase the adhesiveness of vascular endothelial cells and platelet aggregation, and further promote thrombus formation. Moreover, EVs can also activate inflammatory responses and immune responses, leading to inflammation and damage in the vascular wall, further promoting the development of VTE. In patients with PH, it was found that the content of EVs in the blood and lung tissue was significantly increased. These EVs may arise from multiple cell types, including pulmonary artery endothelial cells, smooth muscle cells, and inflammatory cells. EVs can influence the pathogenesis of PH through several mechanisms by carrying bioactive molecules such as inflammatory factors, miRNA and cytokines. Moreover, EVs can also affect vascular remodeling and pulmonary artery constriction by regulating the function of vascular endothelial cells.

In this review, we summarize the current knowledge concerning the role of EVs in two pathophysiologic conditions that can sometimes be linked and co-exist in the same subjects: VTE and PH. We aim to determine the similarities and distinctions between these two diseases and to discuss the pathophysiologic roles of EVs as well as their potential as biomarkers for VTE and PH. For simplicity, this review will refer to both MVs and exosomes presented in published studies as EVs.

## Mechanistic contribution of EVs in the pathophysiology of VTE

Thus far, many studies have confirmed that circulating EVs are increased in VTE patients, suggesting that EVs might be involved in the pathophysiology of VTE [16–20].

### EVs and hypercoagulability or thrombosis

There is a close correlation between EVs and hypercoagulability or thrombosis. EVs are involved in the regulation of hypercoagulability and thrombosis by carrying and releasing blood coagulation-related molecules, and by interacting with target cells such as platelets, leukocytes and endothelial cells.

All circulating EVs display inherent procoagulant properties as they provide an anchoring surface membrane for the assembly of components from the coagulation cascade [21]. Furthermore, the potential for promoting coagulation is increased by the exposure of negatively charged membrane glycerophospholipids, particularly phosphatidylserine (PS), and the expression of key tissue factor (TF) involved in the coagulation cascade [22, 23]. Transmembrane glycoprotein TF, as a receptor for FVII/VIIα, is the primary initiator of coagulation in vivo. Subsequently, the TF/FVIIα complex activate both FX and FIX to facilitate thrombin formation [24]. Circulating EVs bearing TF mainly originated from monocytes and tumor cells [25–27] thus contributing to the greater incidence of VTE associated with cancer. Although circulating TF-positive EVs are normally relatively low in number in physiological conditions, their number rises in pathological conditions, particularly cancer and infectious diseases. In addition, the density of TF on EVs has been shown to be higher than at the surface of their parental cells [27]. Furthermore, PS that resides in the inner leaflet of membrane bilayer can increase the ability of TF to initiate coagulation [20]. In contrast, cells activation or apoptosis lead to the translocation of PS to the outer leaflet of the membrane bilayer and increase membrane blebbing, the forming of irregular bulge(s) in the cell membrane by localized decoupling of the cytoskeleton from the cell membrane, and subsequent EVs shedding. Indeed, the exposure of PS at the membrane surface facilitates the electrostatic interaction with the electropositive γ-carboxyglutamic acid (GLA) domains in the clotting proteins (FVII, X, IX and II) [28]. In addition, PS accelerates the formation of the tenase and prothrombinase complexes, ultimately promoting thrombin formation [19].

Of note, circulating EVs mainly originate from platelets, red blood cells, leukocytes, and endothelial cells. EVs of different origins and released in different pathophysiological conditions may have different procoagulant activities.

### Platelet-derived EVs

As the most abundant EVs in human body, platelet-derived EVs (PEVs) account for 70–90% of circulating EVs in the blood [29, 30]. PEVs express PS, TF and receptors for coagulation factors at their surface membranes, thus inducing procoagulant properties [31]. PEVs membranes exhibit a procoagulant activity 50- to 100-fold higher than in activated platelets [32]. P-selectin, CD40 and CD40 ligands (CD40L or CD154) at the surface of activated platelets can interact with their respective receptors presented in leukocytes (P-selectin glycoprotein ligand-1 (PSGL-1) or CD40). This interaction induce the upregulation of leukocytes-derived TF, thereby promoting the thrombotic progression [33]. Therefore, Lin et al. proposed that these adhesive molecules presented at the surface of PEVs are also important for monocyte aggregation [31]. They further indicated that PEVs can trigger cell-mediated thrombogenicity and lead to the generation of pro-coagulant TF-bearing EVs secreted by monocytes [31].

In addition to facilitating thrombin formation by presenting a procoagulant surface factors, PEVs and red blood cell-derived EVs (RBC-EVs) can initiate the formation of thrombin in a FXII-dependent manner in vitro [34] and in FXI-dependent manner in human endotoxemia [35]. In an experimental model of venous thrombosis in mice, Ramacciotti et al. found a positive correlation between PEVs infusion and thrombus weight, while in contrast the infusion of leukocyte-derived EVs (LEVs) negatively correlated with thrombus weight [36]. Therefore, they hypothesized that PEVs may be a marker for thrombosis in this experimental model, while LEVs were involved in thrombus resolution.

Thrombin can activate platelets and also promote the release of PEVs. As it was shown that thrombin-stimulated platelets produced 2.71 times more PEVs than unstimulated platelets in vitro [37]. FBXW7 (F-Box and WD repeat domain containing 7) and EFNA1 (Ephrin A1) were reported to be able to regulate endothelial barrier function and endothelial proliferation [38, 39]. After being activated by thrombin, platelets can release a large amounts of the miR-223 in PEVs, and PEVs then deliver functional Ago2 miR-223 complexes to human umbilical vein endothelial cells (HUVECs) in turn reducing the endothelial expression of its mRNA targets FBXW7 and EFNA1, at the transcriptional and protein level, this can further promote endothelial dysfunction. Furthermore, miR-223 in PEVs secreted by platelets can facilitate endothelial cells apoptosis by downregulating the expression of vascular insulin-like growth factor 1 receptor (IGF-1R) and promote thrombin formation [40].

### Red blood cell-derived EVs

Erythrocytes spontaneously produce PS-positive EVs during their maturation. Under storage conditions for transfusion, erythrocytes release more EVs [41]. However, in a proteomic analysis of RBC-EVs, TFs were not identified and their ability to increase thrombosis generation has been shown even at alow expression level or absence of TF [42, 43]. Moreover, it has been mentioned earlier that RBC-EVs can promote thrombin generation in a FVII-dependent manner [34]. Furthermore, RBC-EVs accelerate the generation of thrombin through the interaction with FXI in human endotoxemia [35], sickle cell disease [43] and donor blood units samples [44]. Von Willebrand factor (vWF) is a macromolecular protein produced by platelets and endothelial cells. vWF plays an important role in the process of blood clotting by facilitating platelet aggregation and the initiation of the clotting process [45]. Straat et al. proposed that RBC-EVs are the main source of von Willebrand factor (vWF), which takes a part in both cell adhesion and thrombin formation [46].

### Monocyte-derived EVs

Monocyte-derived EVs (MEVs) exhibit both PS and TF at their membrane surface and the prothrombinase activity of TF-positive MEVs was 2.8-fold higher than for TF-negative PEVs [47]. As for PEVs, MEVs were confirmed to modulate blood clot formation in vitro [48, 49], although it might be different from thrombus formation in vivo. In pathological conditions, increased circulating MEVs play a more significant role in thrombin formation [50].

MEVs can increase fibrin network density and increase clot resistance to fibrinolysis [47]. Additionally, MEVs could also express adhesive molecules such as CD15 or PSGL-1, thereby enabling the docking to activated platelets by binding to P-selectin and leading to TF accumulation in developing thrombus and fibrin formation [51]. Finally, TF-positive MEVs can also interact with neutrophils and stimulate their procoagulant activity [52].

### Endothelial cell-derived EVs

Endothelial-derived EVs (EEVs) bearing both TF and PS are released when endothelial cells (ECs) were activated. EEVs play a vital role in thrombogenesis by binding to monocytes [53, 54]. EEVs interact with monocytes through the interplay of ICAM-1 on EEVs and β2 integrins on monocytes, stimulating TF expression by monocytes [55]. Additionally, EEVs depend on the expression of super-large vWF to promote and stabilize platelet aggregates [56]. Oxidized phospholipids in EEVs exposed to oxidative stress might be particularly active in mediating both monocyte adherence to ECs and the activation of neutrophils, which are involved in thrombus formation [57, 58].

### EVs in endothelial dysfunction

The vascular wall damage component from the initial Virchow’s triad is now more broadly recognized as endothelial dysfunction (ED). We now know that the vascular ECs critically contribute to the maintenance of cardiovascular homeostasis by releasing vasoconstrictors and vasodilators and by regulating inflammation and thrombosis. ED is an initiating event in the occurrence and development of cardiovascular diseases [59]. In addition, it is regarded as an indispensable contributor in the development of VTE. ED is characterized by disruption of NO production, inflammation and coagulation, which ultimately influences angiogenesis and apoptosis. During ED, numerous functions of ECs are disturbed and increasing evidence in the literatures have established the potential role of circulating EVs in the development of ED [59–61].

### Platelet-derived EVs

PEVs together with abnormal shear stress can stimulate ECs in turn secreting cytokines and enhancing ICAM-1, VCAM-1and E-selectin expression [62]. PEVs containing arachidonic acid can activate adjacent platelets and ECs [63]. CCL5 (RANTES) is a cytokine that regulates cell activity by binding to specific cell surface receptors. PEVs can deliver CCL5 (RANTES) to the inflamed and atherosclerotic endothelium, triggering monocytes adhesion [64].

Importantly, most of the miRNAs in human plasma was localized in EVs [65]. Diehl et al. demonstrated that the predominant amount of plasma miRNA was associated with EVs and only small amounts were detected in EVs-free plasma underlining the importance of EVs as miRNA transport vesicles in circulation [65]. They found that plasma preparations contained 5200 EVs/uL, 41 to 45% of circulating EVs were from platelet, 28% from leucocyte, and 8% from endothelial origin. PEVs are seen as important vesicles to transfer platelet-specific miRNAs to ECs. Diehl et al. found that activated platelets can release functional miRNAs to promote ICAM-1 expression in ECs [66]. Bao et al. found that miR-142-3p and miR-223-3p were increased in PEVs in hypertension [37]. In vitro studies showed that PEVs containing BCLAF1-targeting miR-142-3p promoted ECs proliferation via BCL2 and Bax. In addition to promoting thrombin formation, miR-233 can induce vascular ECs apoptosis by targeting IGF-1R [40]. Another in vitro study however found that PEVs could reduce ECs activation. Lee et al. found that PEVs transferred into ECs can inhibit the expression of ICAM-1 in TNF-α-stimulated HUVECs by regulating the MAPK and NF-κB signaling pathways through miR-223 [67].

### Red blood cell-derived EVs

RBC-EVs are structurally and functionally distinct from their parenting RBCs [68]. RBC-EVs contain a significant amount of hemoglobin (Hb), which confers to RBC-EVs physiological properties that are closer to cell-free Hb than intact RBC, especially in terms of interactions with nitric oxide (NO) [41]. NO was considered as the endothelial-derived relaxing factor, modulating vascular tone and reducing platelet activation and cellular vascular adhesion [69]. Gladwin et al. found that RBC-EVs can scavenge NO many times faster than RBC [70] in relation with higher Hb concentration in smaller RBC-EVs [71]. Moreover, RBC-EVs are more accessible to close to ECs, subsequently scavenge NO [41].

Straat et al. found that monocytes binding or phagocytosing RBC-EVs upregulate ICAM-1 and E-selection expression in ECs, and this process is mediated by β-integrin [46]. However, RBC-EVs alone or co-incubated with neutrophils cannot activate ECs, suggesting that the effect was specific for monocytes.

### Monocyte-derived EVs

Various monocytes stimulation can alter EVs cargo but not their size or concentration [72]. LPS-stimulated MEVs can promote the expression of ICAM-1, CCL2, and IL-6 through an NF-κB mechanism involving TLR4. In another study, the same stimulus promoted ICAM-1, VCAM-1, and E-selectin expressions by the ERK1/2 and NF-κB pathways [73]. MEVs are capable of autocrine activation, inducing the production of superoxide anions, the release of cytokines and the activation of NF-κB, ultimately leading to EC dysfunction [74]. Apoptotic MEVs enhance the release of NO from ECs. This process involves the activation of PI3-kinase and ERK1/2 promoting the expression of caveolin-1 albeit not its phosphorylation. Interestingly, MEVs treatment leads to an increase in protein nitration at the tyrosine residue participating in ED [75].

### Endothelial cell-derived EVs

TNF-α-stimulated EEVs interact with ECs to induce the expression of IL-10 [72], ICAM-1 as well as pro-apoptotic molecules [76] leading to monocytes adhesion. EEVs produced under pathologic hyperglycemic conditions show increased NADPH oxidase activity and contain higher levels of ROS. TNF-α-stimulated EEVs stimulate endothelial inflammation through a NADPH oxidase-ROS-p38 signaling pathway and upregulate ICAM-1 and VCAM-1 expression as well as promote monocyte adhesion [77]. Activated EEVs by high glucose and AngII aggravated ED by increasing ERK1/2 signaling and reducing eNOS protein expression level in mice aortas [78]. Zernecke et al. found that miR-126 enriched in apoptotic bodies can interact with adjacent ECs and decrease atherosclerosis in mice [79]. However, Jansen et al. found that EEVs containing miR-222 reduced ED through the downregulation of ICAM-1 [80]. The apparent conflictual role of EEVs may partly relate to the specific pathophysiologic conditions and the known specialization of EVs, ECs of different origins within different conditions produce EVs with different contents and functions [81].

### EVs and impaired blood flow

PS-positive EVs with or without TF are mostly cleared by the spleen [82–84]. Reduced blood flow promotes EVs accumulation at the endothelial surface and reduce the clearance of circulating EVs. Furthermore, venous blood stasis leads to hypoxia in the valvular pockets of large veins where venous thrombi are generated. Hypoxia can in turn lead to ED with increased EVs production contributing thrombin formation [11].

### Mechanistic role(s) of EVs in the pathophysiology of PH

Numerous studies found that PH patients exhibit higher levels of circulating EVs than healthy subjects [85–91]. Further studies found that EVs play a crucial role in the pathogenesis of PH [92].

Healthy mice injected with EVs harvested from the plasma or lung tissues of mice with monocrotaline-induced PH (MCT-PH) subsequently developed PH [93, 94]. Blair et al. have found that circulating EVs from late stage PH rats stimulated ICAM-1 expression in the pulmonary arterial endothelium but not in the pulmonary microvascular endothelium [95]. These results were consistent with previous report supporting molecular and functional heterogeneity in pulmonary ECs [96]. However, whether EVs can induce the expression of other inflammatory molecules in the microvascular endothelium or not needs further studies. In addition, the EVs isolated from MCT-PH mice were mainly derived from pulmonary ECs [93]. There were no differences in the number of lung tissue-derived EVs and blood plasma-derived EVs isolated from MCT-injured or vehicle-treated mice, but qualitative differences were observed [93].

Indeed, lung-derived EVs from MCT-induced PH, but not plasma-derived EVs, showed higher and different endothelial mRNA expression such as PDGF, BNPR2 and eNOS. Furthermore, miRNA microarray analysis was performed on EVs-based miRNAs, and found that EVs from both MCT-PH rats and PH patients contained elevated miRNAs, which may contribute to the development of PH [97]. The types of miRNAs commonly increased in EVs derived from MCT-PH rats and IPAH patients were found to include miR-17–92 cluster, miR-21, and miR-145. Previous studies have suggested their roles in pulmonary vascular remodeling although the underlying mechanisms linking these miRNA and the development of PH remains poorly known and require further studies to evaluate their potential for treatment of PH. Interestingly, the resource of EVs was mainly from platelets and erythrocytes in hypoxia-induced PH mice [98]. Circulating EVs generally refer to the extracellular vesicles that are found in the bloodstream or other bodily fluids. Circulating EVs derived from platelets and red blood cells in blood of hypoxia-induced PH mice can induce ED by both decreasing NO production and increasing oxidative stress in pulmonary EC [98].

As for VTE, hypercoagulability and ED may partly contribute to the pathogenesis of PH [92]. In the following section of this review, we will summarize the role of EVs on pulmonary arterial smooth muscle cells (PASMCs) and the role of EVs as a mean of crosstalk between pulmonary arterial endothelial cells (PAECs) and PASMCs in the development of PH.

### Endothelial cells-derived EVs

Previous research has confirmed that ED could lead to an increased PAECs-derived EVs secretion, which transferred to PASMCs to induce its over-proliferation and conversion to an apoptosis-resistant phenotype [99]. In addition, PAECs-derived EVs miR-210-3p contribute to the proliferation of PASMC and the development of PH induced by hypoxia [100]. Su et al. have demonstrated that miR-1249 from PAECs-derived EVs mediated cigarette smoke-induced PH through the inhibition of the HDAC10-NFκB-CaSR cascade [101]. Besides miRNA, spermine enrichment at the outer surface and cytosol of PAECs-derived EVs mediated smoking-induced PH partially through CaSR [102]. However, EVs may also exert a protective role in PH. EVs can either promote or prevent PH based on their content. Endothelial cells-derived EVs reduce the proliferation and migration of vascular smooth muscle cells (VSMCs), and subsequent neointima formation by delivering functional miR126 into recipient VSMCs. In addition, endothelial cells-derived EVs transferred miR195 to SMCs, and miR-195 inhibit the proliferation of SMCs through 5-HTT [103]. Krüppel-like factor 2 (KLF2) is a transcription factor and plays a critical role in the regulation of lung function and development when it is activated by shear stress [104]. Sindi et al. found that KLF2-induced PAECs-derived EVs attenuate pulmonary vascular remodeling through the combined action of miR-181a-5p and miR-324-5p [104]. ECs overexpressing KLF2 induced the production of miRNA143/145 that are then delivered to VSMCs via EVs and depress the target genes expression to reduce atherosclerosis, and these target genes come from the research reports and target prediction including *ELK1* (ETS transcription factor ELK1), *KLF4* (Kruppel-like factor 4), *CAMK2d* (calcium/calmodulin-dependent protein kinase type II subunit delta), *CFL1* (cofilin 1), *PHACTR4* (phosphatase and actin regulator 4), *SSH2* (slingshot protein phosphatase 2) and *MMP3* (matrix metallopeptidase 3) [105].

### Smooth muscle cells-derived EVs

Zheng et al. have found that smooth muscle cells (SMCs) exposed to oxLDL enhance their expression of KLF5 that in turn reduced miR143/145 and miR211/222 expression while increasing miR155 and miR146a expression. EVs-mediating miR155 transfer from SMCs to ECs changed the endothelial barrier by inhibiting the expression of the tight junction protein, especially ZO-1, consequently increasing microvascular permeability and promoting atherosclerosis [106]. PASMC-derived EVs were internalized by PAECs and the mRNA cargo transfer can promote the endothelial-to-mesenchymal transition in HPAECs contributing to the development of PH [107]. MiR-221/222 as content of Human aortic smooth muscle cells-derived exosomes inhibited the autophagy of HUVECs by inhibiting the expression of PTEN and subsequently activating Akt signaling [108]. EVs containing miR143 derived from PASMCs may be transferred to PAECs to facilitate ECs migration and angiogenesis [109].

### Other cells-derived EVs

Other sources of EVs are involved in the vascular remodeling as well. PEVs firmly bound to resting SMCs to facilitate the adhesion of monocyte to SMCs and induce the secretion of IL-6 leading to a pro-inflammatory SMCs phenotype and stimulating vascular remodeling [110]. As a scavenger receptor, the lectin-like oxidized low-density lipoprotein receptor-1 (LOX-1) can promote endothelial dysfunction and contribute to the development of atherosclerotic cardiovascular disease [111]. A recent research has found that plasma-derived EVs can drive hypoxic PH by transferring LOX-1 cargo to trigger phenotypic switching of PASMCs from contractile phenotype to proliferative phenotype [112]. MEVs may transfer caspase-1 into the cytosol of ECs and SMCs in turn inducing apoptosis [113]. Sharma et al. have demonstrated that EVs derived from HIV-infected monocyte-derived macrophages exposed to drugs of abuse can induce the proliferation of human PASMCs [114].

## The differences in the role of EVs in VTE and PH

As mentioned above, EVs play important roles in VTE and PH. However, due to the differences between VTE and PH pathogenesis, there are different roles played by EVs in these two diseases. The differences may be related to the underlying mechanism and clinical manifestations of both diseases, and further investigation of the mechanism of action and regulatory pathway of EVs are important for an in-depth understanding of the occurrence and development of VTE and PH.

In VTE, the critical role of EVs is thought to promote thrombosis and ED [16]. It has been shown that EVs can release platelet-activating factors and coagulation factors during thrombus formation, thus promoting the occurrence of platelet aggregation and coagulation cascade [33, 46, 50, 56]. Moreover, EVs can also activate ECs, leading to the development of vascular ED and the occurrence of inflammatory responses, further promoting thrombosis [62, 63, 74].

In contrast, in PH, the role of EVs is much more complex and diverse. Studies have shown that increased EVs release exists in the circulatory system of patients with PH [81]. These EVs can carry a variety of bioactive molecules, such as cytokines, growth factors and miRNA, and participate in the development and development of PH by interacting with target cells [100, 102, 104, 112]. First, EVs can promote pulmonary vascular contraction and pulmonary vascular remodeling by activating the SMCs and ECs of the pulmonary arteries, thus leading to the development of PH [107]. Second, EVs can also participate in the pathological process of PH by regulating the immune response and inflammatory response.

## EVs as biomarkers for VTE and PH

A large number of previous studies have measured and compared the levels of EVs originating from different cell types in patients versus healthy subjects and recommended EVs as a biomarker in VTE and PH. The recognition of EVs as biomarkers for VTE have been elegantly reviewed in 2012 by Rautou et al. [19]. Hence, Tables 1 and 2 recapitulate the major findings highlighting the place of EVs as biomarkers for VTE from 2012 up-to-date for VTE and since 2008 for PH.

### EVs (endothelium, platelet, monocyte, etc.) and TF-EVs as a biomarker for VTE

See Table 1

**Table 1:** Studies measuring EVs in patients with venous thromboembolism

Disease (number of patients/controls)	method	Main findings	References
Patients: 117 with PBC Pancreatic (n = 80) Biliary (n = 34) Unknown primary histologically consistent with PBC (n = 3)	Functional assay (MP-TF activity)	36 (36/117) patients experienced VTE Elevated circulating TF^+^EVs activity is associated with VTE TF^+^EVs were higher in patients with VTE versus those without VTE	[115]
Patients: 252 with various cancers Healthy controls (n = 36)	ELISA and chromogenic assays	34 (34/252) patients experienced VTE There was no statistical association between TF^+^EVs activity and the VTE risk	[116]
Patients with GBM (glioblastoma multiforme) (n = 61)	FCM (flow cytometry)	11 (11/61) patients experiencing VTE have higher level of TF^+^EVs than those without VTE The levels of TF^+^EVs above the 90% were associated with higher VTE risk	[117]
Acute DVT patients (n = 41) Healthy control (n = 10)	Functional assay ((MP-TF activity)	The activity of TF^+^EVs was low at the acute event but remained unchanged during a follow-up period	[118]
Soft tissue sarcoma patients (n = 39) Healthy controls (n = 17)	FCM	In patients with VTE, significantly higher levels of activated(CD62P^+^CD63^+^) PEVs were found compared to patients without VTE	[119]
Unprovoked VTD patients(n = 138) Advanced cancer patients(n = 67)	FCM	TF^+^EVs were significantly increased in patients with unprovoked VTD compared to cancer patients without thrombosis	[120]
Patients with unprovoked VTD (n = 40) Healthy controls (n = 40)	Prothrombinase assay	PEVs were higher in DVT in comparison with healthy controls	[121]
Patients with glioma (n = 11) Healthy controls (n = 10)	FCM	In patients with GM, the relative abundance of CD41^+^PEVs and CD105^+^EEVs was significantly higher than in the control group	[122]
Trauma Patients With DVT(n = 53) Trauma Patients Without DVT(n = 53) Healthy control (n = 53)	FCM	hepatocyte-derived MPs (HMPs) and phosphatidylserine (PS)^+^ HMPs were significantly increased in trauma patients with DVT, but lower in trauma patients without DVT	[123]

*PBC* pancreaticobiliary cancer, *VTE* venous thromboembolism, *PE* pulmonary embolism, *GBM* glioblastoma multiforme, *DVT* deep vein thrombosis, *FCM* flow cytometry

### EVs (endothelium, platelet, etc.) as a biomarker for PH

See Table 2

**Table 2:** Studies measuring EVs in pulmonary hypertensionpatients

Disease (number of patients/controls)	Method	Main findings	References
Patients undergoing right heart catheterization for precapillary PH without any endothelium-active vasodilator therapy (n = 24) Healthy controls (n = 20)	FCM	Levels of circulating CD31^+^/CD41^−^, CD144^+^, CD62e^+^ EEVs and CD45^+^ EVs, but not CD41^+^ and annexin^+^, were increased in PH patients compared with healthy controls CD31^+^/CD41^−^ and CD144^+^ EEVs levels predicted hemodynamic severity of PH CD62e^+^ EEVs were associated with inflammation	[91]
IPAH patients (n = 20) PAH patients with CTD(n = 5) PAH patients with HIV infection (n = 2) PAH patients with portal hypertension (n = 2) Healthy controls (n = 23)	Solid-phase capture assay	TF^+^ and CD105^+^EVs were increased in PAH patients compared with control CD105^+^EVs were increased in pulmonary arterial blood compared with venous blood TF^+^EVs were at a higher level in patients in functional class III and IV and who were walking fewer than 380 m with the six-minute-walk test	[88]
Patients undergoing right heart catheterization for untreated pre-capillary PH (n = 21)	FCM	Elevated circulating CD62e^+^ EVs levels in patients with PH before treatment are associated with adverse clinical events	[87]
PHpatients with OSA (n = 7) PH patients without OSA (n = 18)	FCM	There were no statistical significant differences regarding EEVs or other EV group levels, including the apoptotic AnnexinV^+^ EVs, between PH patients with and without OSA	[124]
CHD with reversible PAH (n = 16) CHD with irreversible PAH (n = 10)	FCM	There was no significant difference in the levels of (CD31^+^/CD41^−^) EEVs between CHD with reversible and irreversible PAH	[125]
Chromic postembolic PH (n = 19) IPAH (n = 5) Rheumatic disease associated PH (n = 4) Pulmonary diseases related PH (n = 2) Healthy controls (n = 16)	FCM	PH patients have increased levels of platelet-( CD31^+^/61^+^), leukocyte- (CD11b^+^) and endothelial (CD62e^+^) EVs	[90]
patients with advanced IPAH(n = 10) Healthy controls (n = 10)	FCM	circulating (CD39^+^CD31^+^CD42b^+^) PEVs and (CD39^+^CD31^+^CD42b^−^) EEVs subpopulations in IPAH patients show increased CD39 expression	[126]
hPAH patients (n = 18) IPAH patients (n = 19) aPAH patients (n = 17) Healthy controls (n = 21)	FCM	EEVs and small PEVs levels in IPAH, hPAH and aPAH were similar and significantly increasedcompared with controls	[127]
PAH patients (n = 22) Healthy controls (n = 20)	FCM	EEVs in urine were increased in PAH compared tohealthy controls EEVs were directlyassociated with TAPSE in PAH patients	[128]
CTEPH patients (n = 6) PE patients (n = 6) Healthy controls (n = 4)	FCM	CD105^+^EVs were higher in CTEPH patients than in PE patients and healthy controls CD105^+^ EVs exerts a protective mechanism that supports survival of ECs and angiogenesis	[89]
SSc patients(n = 10) SSc associated PAH (n = 10) Healthy controls (n = 10)	FCM	CD144^+^ EEVs were significantly higher in the SSc-PAH group compared to the SSc patients or healthy controls	[129]
PAH patients (n = 70) Healthy controls (n = 20)	FCM	CD62P^+^, CD144^+^, and CD235a EVs were higher in PAH blood compared to healthy controls	[86]
IPAH patients (n = 24) aPAH patients(n = 8) Porto-pulmonary hypertension associated PAH (n = 6)CHD associated PAH (n = 3) pulmonary veno-occlusive disease/pulmonary capillary hemangiomatosis (n = 3) CTEPH (n = 29)	ELISA	Increased PEVs (CD42a, CD42b) have been shown in IPAH patients, which were higher in male patients	[130]
PAH patients (n = 144) Healthy controls (n = 47)	FCM	PAH patients showed a significantly higher numbers of CD31^+^/CD42b^−^EEVs compared with healthy controls	[131]

*CTEPH* patients with chronic thromboembolic pulmonary hypertension, *PE*: Pulmonary embolism, *IPAH* idiopathic pulmonary arterial hypertension, *hPAH* hereditary PAH associated with BMPR2 (Bone morphogenetic protein receptor, type II) mutation, *aPAH* PAH associated with CTD (connective tissue diseases), *FCM* Flow cytometry, *TAPSE* Tricuspid annular plane systolic excursion, *OSA* obstructive sleep apnea, *CHD* congenital heart disease

## EVs as therapeutic application for VTE and PH

Extracellular vesicles can be propagated to different tissues and organs via blood circulation and interact with target cells. Thus, the use of extracellular vesicles for disease therapy or as carriers to deliver therapeutic agents has great potential.

There are evidences that bone marrow-derived endothelial progenitor cells (EPCs) can contribute substantially to the resolution of thrombosis [132]. Previous studies have demonstrated that miR-126 can improve endothelial progenitor cells migration and angiogenesis, and promote the recruitment of endothelial progenitor cells in venous thrombosis [133]. Sun et al. found that translocation of miR-126 into EPCs-derived EVs by electroporation can promote dissolution and recanalization of DVT [134]. Furthermore, PEVs carrying miR-320 can relieve inflammation and thrombosis by promoting the mobility of ECs [66]. PEVs carrying miR-223 can also inhibit ICAM-1 expression in ECs during inflammation [67].

Numerous studies have shown that mesenchymal stem cell (MSC)-derived EVs can be used as a therapeutic strategy for the improvement of PH. Chen et al. demonstrated that intravenous injection of MSC-derived EVs significantly improved MCT-induced rat PAH[135]. Aliotta et al. also confirmed that MSC-derived EVs could reverse progression of MCT-induced mice PAH [136]. It has been shown that treatment with MSC-derived EVs can significantly inhibit vascular remodeling in PAH patients by reducing PASMCs proliferation and right ventricular systolic as well as pulmonary arterial systolic pressure [137]. MSC-derived EVs have also been proved to reverse the development of PH in mice treated with EPCs from PAH mice [138]. In addition to MSCs, it has been reported that endothelial cells-derived EVs transferred miR195 to SMCs can prevent the proliferation and migration of SMCs and contribute to the improvement of PH [103].

## Future challenges

EVs play critical roles in VTE and PH, mainly involved in the pathogenesis of both diseases, and are potentially suitable to be diagnostic markers and therapeutic targets. However, EVs from different sources vary widely in composition and cargo. Most of the studies included in this review mainly investigated the role of miRNAs as the cargo of EVs in the pathogenesis of VTE and PH. However, studies on other important EVs content such as protein, lipids and DNA are lacking and require further research and exploration. The field of EVs research expands very rapidly, but due to the inherent characteristics of EVs, there are remaining challenges with their use in the clinical detection of disease activity as well as therapy. The current methods used to isolate and analyze EVs lack consistency and a standardized approach with quality controls of EVs preparation is required. The content of EVs varies considerably from the parent cells and EVs selection should be designed according to the desired target molecules of the recipient cells. Moreover, differences in the complexity and severity of the disease may have some impact on EVs as biomarkers and therapeutic approaches. Despite these limitations, further identification of EVs and corresponding cargo can help to discover clinically useful biomarkers and develop new treatments for disease.

## Conclusion

We have reviewed the known potential roles of EVs in two pathophysiologically linked conditions, VTE and PH. In both conditions there is solid evidence that EVs contribute to vascular endothelial dysfunction, inflammation, thrombosis and cellular activation and communications. The roles and importance of EVs substantially differ between studies depending on experimental conditions and parent cell origins of EVs that modify the nature of their cargo. Animal models have clearly established that EVs contribute to the pathophysiology of VTE and PH. Human studies have found increased levels of various EVs in relation with the severity of VTE and PH, confirming its potential pathophysiological role and its utility as a biomarker of disease activity and severity. However, we failed to identify clear disparities between EVs in VTE versus PH such that a distinctive signature is currently not evident. Further research should help to delineate the distinctions between EVs in VTE versus PH to develop more specific markers of disease and exploring potential novel therapeutic targets.

## Data Availability

Not applicable.

## Abbreviations

EVs: Extracellular vesicles
MVs: Microvesicles
MPs: Microparticles
PM: Plasma membrane
MVBs: Multivesicular endosomes or bodies
VTE: Venous thromboembolism
PH: Pulmonary hypertension
DVT: Deep vein thrombosis
PE: Pulmonary embolism
PS: Phosphatidylserine
TF: Key tissue factor
PEVs: Platelet-derived EVs
PSGL-1: P-selectin glycoprotein ligand-1
RBC-EVs: Red blood cell-derived EVs
LEVs: Leukocyte-derived EVs
HUVECs: Human umbilical vein endothelial cells
vWF: Von Willebrand factor
MEVs: Monocyte-derived EVs
EEVs: Endothelial EVs
ED: Endothelial dysfunction
Hb: Hemoglobin
MCT-PH: Monocrotaline-induced PH
PASMCs: Pulmonary arterial smooth muscle cells
PAECs: Pulmonary arterial endothelial cells
EPCs: Endothelial progenitor cells
